# Volunteering and Cardiovascular Biomarkers: A Machine-Learning Causal Approach in Health and Retirement Study

**DOI:** 10.1093/geroni/igaf122.203

**Published:** 2025-12-31

**Authors:** Seoyoun Kim, Koichiro Shiba, Cal Halvorsen

**Affiliations:** University of Michigan, Ann Arbor, Michigan, United States; Boston University, Boston, Massachusetts, United States; Washington University in St. Louis, St. Louis, Missouri, United States

## Abstract

Prosocial engagement, such as volunteering, has been recognized for its protective effects against cardiovascular diseases (CVD), yet its causal impact on CVD-related biomarkers remains unclear. This study employs targeted maximum likelihood estimator (TMLE), a doubly robust causal inference method, to estimate the effect of the changes in volunteer activity (2006/2008 and 2010/2012) and seven key CVD-related biomarkers (in 2014/2016) using the Health and Retirement Study (N = 17,479). The biomarkers assessed include blood glucose (HbA1c), lipid regulation (TC/HDL ratio), chronic inflammation (CRP), kidney function (Cystatin C), blood pressure (systolic and diastolic), and BMI. Volunteering status was categorized into non-volunteers, initiators, stoppers, and sustained volunteers. TMLE results indicate that, had the entire population engaged in volunteering, we would expect improvements in CVD biomarkers compared to no volunteering at either wave. Sustained volunteering was associated with a 4.6% lower prevalence of unhealthy HbA1c levels (-0.046, p = 0.016), a 1.7% lower prevalence of an unhealthy TC/HDL ratio (-0.02, p = 0.001), an 8.1% lower prevalence of elevated CRP levels (-0.04, p = 0.0003), a 3.7% lower prevalence of unhealthy Cystatin C levels (-0.08, p < 0.001), an 8.9% and 8.7% lower prevalences of elevated diastolic and systolic blood pressure, respectively (DBP: -0.09, p<.001, SBP: -0.09, p<.001). However, a modest 1.2% increase in BMI was also observed. These findings highlight the potential of long-term volunteering as a population-level intervention to improve cardiovascular health, underscoring the need for further research to explore underlying mechanisms and long-term clinical benefits.

